# Screening of Key Drought Tolerance Indices for Cotton at the Flowering and Boll Setting Stage Using the Dimension Reduction Method

**DOI:** 10.3389/fpls.2021.619926

**Published:** 2021-07-09

**Authors:** FengLei Sun, Qin Chen, QuanJia Chen, Menghui Jiang, Wenwei Gao, YanYing Qu

**Affiliations:** College of Agronomy, Xinjiang Agricultural University, Ürümqi, China

**Keywords:** cotton, drought resistance indices, membership function value, principal component analysis, drought resistance

## Abstract

Drought is one of the main abiotic stresses that seriously influences cotton production. Many indicators can be used to evaluate cotton drought tolerance, but the key indicators remain to be determined. The objective of this study was to identify effective cotton drought tolerance indicators from 19 indices, including morphology, photosynthesis, physiology, and yield-related indices, and to evaluate the yield potential of 104 cotton varieties under both normal and drought-stress field conditions. Combined with principal component analysis (PCA) and a regression analysis method, the results showed that the top five PCs among the 19, with eigenvalues > 1, contributed 65.52, 63.59, and 65.90% of the total variability during 2016 to 2018, respectively, which included plant height (PH), effective fruit branch number (EFBN), single boll weight (SBW), transpiration rate (Tr) and chlorophyll (Chl). Therefore, the indicator dimension decreased from 19 to 5. A comparison of the 19 indicators with the 5 identified indicators through PCA and a combined regression analysis found that the results of the final cluster of drought tolerance on 104 cotton varieties were basically consistent. The results indicated that these five traits could be used in combination to screen cotton varieties or lines for drought tolerance in cotton breeding programs, and Zhong R2016 and Xin lu zao 45 exhibited high drought tolerance and can be selected as superior parents for good yield performance under drought stress.

## Introduction

Xinjiang is located in Northwest China and belongs to arid and semiarid areas with high evaporation and a general shortage of freshwater resources. The average annual precipitation is only 58 mm and is exceeded by the potential evapotranspiration (Wang et al., 2010). Cotton is the main economic crop in Xinjiang and accounts for more than one-third of the total agricultural area in the region (Wang et al., 2004; Kang et al., 2012). At present, the cotton planting area in Xinjiang accounts for 70% of the national planting area, and the total output accounts for 84% of the national total (source: National Bureau of Statistics). Drought has a wide range of effects on cotton, and related reports indicate that cotton is affected by drought, resulting in a 34% reduction in cotton production (Ullah et al., 2017). Hence, obtaining and breeding new varieties with high yield and strong drought resistance are currently the main breeding targets (Cattivelli et al., 2008).

Drought tolerance is genetically related to various morphological and physiological characteristics of crops. Among abiotic stresses, drought has the greatest impact on cotton growth and development, which severely limits cotton yield and fiber quality (Wiggins et al., 2013). The decrease in boll quantity and weight is the main reason for the decline in seed cotton yield (Sarwar et al., 2012). Soomroo et al. (2011) showed that stomatal conductance is reduced under water stress, and photosynthetic phenotypic values vary greatly among plants, reflecting potential differences in stress responses or regulatory processes such as stomatal conductance, photosynthetic rate, and storage of photosynthetic products (Kramer and Evans, 2011; Soomroo et al., 2011). Zhang et al. (2010) showed that wilting movement leaves of cotton plants can reduce the degree of photosynthesis decline when they are under water stress. Water deficiency affects photosynthesis and affecting chlorophyll (Chl) content by changing the internal structure of chloroplasts (Huseynova et al., 2016).

At present, 30 traits have been proposed as important indicators of the drought tolerance of cotton (Loka et al., 2011). These traits mainly have three types, including morphological and physiological indicators (Zhang et al., 2010; Song et al., 2017), photosynthetic indicators (Lawlor and Cornic, 2002; Flexas et al., 2006), and yield indicators (Hussein et al., 2011; Sarwar et al., 2012). These indicators have been widely used in drought resistance studies of wheat, cotton, and maize (Kramer and Evans, 2011; Soomroo et al., 2011; Wiggins et al., 2013). Scientists have combined the comprehensive drought resistance coefficient, stress sensitivity index, membership function, principal component analysis (PCA) and other methods to evaluate drought resistance from cotton yield (Li et al., 2011; Liu T. P. et al., 2014; Huseynova et al., 2016; Lv et al., 2019).

Drought stress reduces yield because it affects crop growth and physiological metabolism (Nagy et al., 2013; Liu et al., 2015), which includes many drought resistance indicators, and it is difficult to consider all indicators when analyzing macropopulations. However, PCA can be used to determine the weight of each indicator and finds some principal components that can control all variables (Ali et al., 2014; Chen et al., 2014; Wijewardana et al., 2016; Bo et al., 2017; Füzy et al., 2019). It can reduce the number of measurement indicators and improve measurement efficiency. Additionally, joint application PCA, membership function, cluster analysis and other methods will make the assessment of crop stress performance more reliable and practical. Recently, Munir et al. (2020) combined PCA to screen out other morphological parameters associated with increasing seed cotton yield and screened out two varieties with higher yields. The drought tolerance of maize inbred lines was evaluated using methods such as PCA and fuzzy clustering, avoiding the one-sidedness of a single indicator and revealing the relationship between drought tolerance traits and crop drought tolerance (Huseynova et al., 2016). This method is also used to screen drought-tolerant varieties of wheat and bread wheat (Farshadfar et al., 2011; Khalili et al., 2012). Therefore, the objective of this study was to screen the key drought tolerance indicators of cotton through PCA and regression analysis and evaluate the drought tolerance ability of 104 cotton varieties.

## Materials and Methods

### Plant Material Drought Treatment

This study was conducted at the Experimental Farm of the Cotton Breeding Laboratory of Xinjiang Agricultural University Experimental Station (43°20′∼45°20′E, 84°45′∼86°40′N) from 2016 to 2018. The average altitude of the area is 300∼500 m, which is a temperate continental climate. The annual average temperature is 7.5∼8.2°C, the sunshine duration is 2318∼2732 h, the frost-free period is 147∼191 d, the annual precipitation is 125.0∼207.7 mm, the annual evaporation is 1000∼1500 mm, and the monthly average precipitation is 13.0∼20.0 mm. The annual average humidity in 2016–2018 was 72, 71, and 69%, respectively. The soil is mainly sandy loam, which contains 0.23 g/kg available phosphorus, 0.29 g/kg available potassium and 0.33 g/kg total nitrogen, with a pH of 8.3.

Before planting, the plots were divided into two parts in the test area, one for normal watering (CK) and another for drought stress (DS). Each germplasm was planted in two rows 300 cm in length, 25 cm apart and 10 cm between plants for each plot. Drought stress conditions were achieved by manual water control throughout the growing season (stress-treated stop irrigation). In the flowering and boll-forming stage (early July), the control group was watered normally, and the stress group was not treated with water twice. All materials were sampled after two controlled water treatments. A completely randomized block experimental design was used, with three replications for each treatment, each separated by a protective row. One hundred and four cotton varieties were used for this experiment (Appendix Table 1).

### Physiological and Biochemical Traits

In the flowering and boll-forming stage, drought stress conditions were achieved by manual water control (when the soil moisture content dropped by 40%, as shown in Table 1). A portable photosynthesis system (CIRAS-3, United Kingdom) was used to measure photosynthetic indicators (between 10:30 and 12:30 Beijing time in the morning, this time period is the best time for local measurement, avoiding the “photosynthetic lunch break” phenomenon). The functional leaf of each material was used for measurement (the cotton inverted trefoil). The measured indicators include net photosynthetic rate (Pn), stomatal conductance (gs), transpiration rate (Tr), water use efficiency (WUE, WUE = Pn/Tr), intercellular carbon dioxide concentration (Ci), and water vapor pressure deficit (VPD). Three replicates of each species under each treatment condition were used for measurement of the photosynthesis indicators. At the same time, the leaves of the plants used for measuring photosynthetic indicators were used to determine physiological indicators, including malondialdehyde (MDA), Chl and superoxide dismutase (SOD). The test was repeated in triplicate. The MDA content was measured according to the method of Yin et al. (2010). Chl was extracted from leaves using 80% acetone (Lichtenthaler, 1987; Yang et al., 2014). SOD activity was measured by the nitroblue tetrazolium (NBT) method (Zhang et al., 2007). Refer to Appendix 1 for specific methods. The determination of each biochemical index was repeated three times.

**TABLE 1:** Soil water contents during 2016∼2018.

Water contents	2016		2017		2018	
			
	Before stress (%)	In the stress (%)	Before stress (%)	In the stress (%)	Before stress (%)	In the stress (%)
0–20 cm	23.116	13.862	28.801	12.489	21.970	12.170
20–40 cm	24.070	14.901	29.098	12.862	22.980	12.820
40–60 cm	25.137	15.243	29.562	14.725	26.000	14.770
Average	24.108	14.669	29.154	13.359	23.650	13.250

### Morphological and Yield Traits

After maturing in late September, 5 uniformly continuous cotton plants for each variety in each treatment were selected to investigate 8 traits, including plant height (PH), fruit branch number (FBN), effective fruit branch number (EFBN), boll number (BN), effective boll number (EBN), cotton seed yield (CSY), cotton lint yield (CLY), and single boll weight (SBW). The investigation method refers to the “Description Specifications and Data Standards for Cotton Germplasm Resources” (Du and Zhou, 2005).

### Drought Adaptability Analysis

The drought tolerance coefficient of each genotype was calculated by the formula proposed by Blum and Jordan (1985) and Szira et al. (2008). The membership function value of drought resistance (MFVD) was calculated according to the relevant formulas proposed by Chen et al. (2012); Zadeh (1965). The drought tolerant coefficient (DC) was calculated as the ratio of the data derived from the drought stress (DS) and normal watering (CK) treatments of the same accession for each trait using the following equations, and according to DC, MFVD was calculated as:

(1) $$D\,C=\frac{X_{D\,S}}{X_{C\,K}}$$

(2) $$U=\frac{D\,C-{D\,C}_{i\,\min}}{{D\,C}_{i\,\max}-{D\,C}_{i\,\min}}$$

(3) $$M\,F\,V\,D=\frac{1}{n}\,\sum_{i=1}^{n}U$$

X_*DS*_ and X_*CK*_ are the values of the trait for the genotype evaluated under DS and CK treatments, respectively, where *U* is the membership function value of the trait for the genotype for drought tolerance and DC_*imax*_ and DC_*imin*_ are the maximum and minimum values of the drought tolerance coefficient for the trait of all the varieties, respectively.

High-yield classification is the best indicator for assessing drought resistance (Ramirez and Kelly, 1998). Therefore, high yield and high drought resistance were evaluated by the yield reduction value (Yd) under water stress. Control species can reduce environmental factors other than water stress (Golestani-Araghi and Assad, 1998), and the yield reduction value (Yd) was calculated as:

(4) Yd=Yp-Ys

Ys is the yield under water stress, and Yp is the yield under normal irrigation conditions.

### Data Analysis

Nineteen indicators were used for analysis. The data were summarized and calculated using Excel 2010, and each measure of each trait corresponds to the mean of three separate replicates. SPSS software (IBM Inc., Armonk, NY, United States) was used to perform analysis of variance (ANOVA) to test the effects of variety, treatment method and their interaction. Means were compared using the sample t-test. PCA (Stackpole et al., 2011; Chen et al., 2014) was performed using a SPSS 21.0. The hierarchical clustering analysis of MFVD was completed using R (cluster package, version 3.6).

## Results

### Responses of Cotton Various Traits to Drought Stress

To analyze the drought effects of different cotton materials, we investigated 19 drought tolerance-related indicators of morphology, photosynthesis, physiology and yield characteristics under cotton drought stress conditions in three consecutive planting cycles. The results showed that under drought stress, the averages of all 19 traits decreased (Table 2). However, the degree of decline is different under different conditions and in different planting cycles. The coefficient of variation (CV) of the 19 traits was 0.11 to 0.68 under drought conditions and 0.08 to 0.63 under sufficient water conditions. The results indicated that the cotton varieties used in this study had greater variation under drought stress. According to the results of the three-factor analysis of variance, the interaction between different breeds, different treatments, different breeds and different treatments had significant or extremely significant effects on the 19 traits in 3 years (*P* < 0.05 or *P* < 0.01) (Appendix Tables 5–7). And all indicators are extremely significant differences under the two treatment conditions (*P* > 0.01) (Table 2).

**TABLE 2:** Statistics of various traits investigated under two conditions in 3 years.

Year	2016				2017				2018			
			
Treatment	CK		DS		CK		DS		CK		DS	
						
Statistical parameter	Mean ± SD	CV	Mean ± SD	CV	Mean ± SD	CV	Mean ± SD	CV	Mean ± SD	CV	Mean ± SD	CV
PH	60.617.90^a^	0.13	45.245.54^b^	0.12	66.056.36^a^	0.10	58.306.85^b^	0.12	72.629.82^a^	0.14	56.777.34^b^	0.13
FBN	7.360.90^a^	0.12	5.070.85^b^	0.17	7.570.85^a^	0.11	6.280.81^b^	0.13	7.961.08^a^	0.14	6.701.04^b^	0.16
EFBN	5.860.87^a^	0.15	4.020.73^b^	0.18	6.230.84^a^	0.13	5.140.77^b^	0.15	5.511.12^a^	0.20	3.070.98^b^	0.32
BN	8.393.24^a^	0.39	4.681.17^b^	0.25	8.201.44^a^	0.18	6.311.26^b^	0.20	5.961.40^a^	0.23	3.261.18^b^	0.36
EBN	6.581.65^a^	0.25	4.310.99^b^	0.23	7.811.40^a^	0.18	5.891.21^b^	0.21	4.771.47^a^	0.31	2.610.98^b^	0.38
CSY	121.7914.21^a^	0.12	100.0313.23^b^	0.13	112.9714.89^a^	0.13	95.0412.15^b^	0.13	107.6412.22^a^	0.11	94.6013.17^b^	0.14
CLY	51.616.34^a^	0.12	31.876.04^b^	0.19	48.526.87^a^	0.14	29.535.37^b^	0.18	39.495.57^a^	0.14	36.726.57^b^	0.18
SBW	6.090.71^a^	0.12	4.960.74^b^	0.15	5.650.74^a^	0.13	4.750.61^b^	0.13	5.400.57^a^	0.11	4.730.66^b^	0.14
Ci	228.7118.45^a^	0.08	200.4922.44^b^	0.11	261.6746.40^a^	0.18	199.2745.33^b^	0.23	227.6644.99^a^	0.20	158.2352.24^b^	0.33
gs	550.39201.89^a^	0.37	323.22163.29^b^	0.51	166.55105.16^a^	0.63	88.7544.42^b^	0.50	378.14212.05^a^	0.56	130.1075.99^b^	0.58
VPD	2.421.16^a^	0.48	1.510.46^b^	0.30	1.771.00^a^	0.56	1.020.32^b^	0.31	3.690.92^a^	0.25	2.180.59^b^	0.27
Pn	26.236.91^a^	0.26	19.945.63^b^	0.28	8.014.15^a^	0.52	4.581.75^b^	0.38	25.396.85^a^	0.27	11.824.86^b^	0.41
Tr	6.421.15^a^	0.18	4.431.27^b^	0.29	1.771.09^a^	0.62	1.120.28^b^	0.25	6.151.22^a^	0.20	3.930.98^b^	0.25
WUE	4.941.17^a^	0.24	3.600.68^b^	0.19	14.1135.63^a^	2.53	3.851.29^b^	0.34	4.912.88^a^	0.59	2.870.9^b^	0.31
MDA	99.9931.56^a^	0.32	60.0822.78^b^	0.38	161.2536.16^a^	0.22	110.0432.28^b^	0.29	117.1629.92^a^	0.26	77.6622.07^b^	0.28
a	2.981.14^a^	0.38	1.860.90^b^	0.48	2.360.57^a^	0.24	1.520.60^b^	0.39	7.731.54^a^	0.20	5.851.36^b^	0.23
b	1.300.48^a^	0.37	0.740.35^b^	0.47	1.600.52^a^	0.33	0.740.41^b^	0.55	5.131.05^a^	0.20	3.880.93^b^	0.24
Chl	4.061.47^a^	0.36	2.601.11^b^	0.43	3.900.96^a^	0.25	2.541.11^b^	0.44	12.832.59^a^	0.20	9.732.22^b^	0.23
SOD	5.261.32^a^	0.25	4.681.24^b^	0.26	19.667.00^a^	0.36	10.717.28^b^	0.68	0.780.20^a^	0.26	0.520.16^b^	0.31

*Plant height (PH), fruit branch number (FBN), effective fruit branch number (EFBN), boll number (BN), effective boll number (EBN), cotton seed yield (CSY), cotton lint yield (CLY), single boll weight (SBW), photosynthetic rate (Pn), stomatal conductance (gs), transpiration rate (Tr), water use efficiency (WUE), intercellular carbon dioxide concentration (Ci), water vapor pressure deficit (VPD), malondialdehyde (MDA), chlorophyll (Chl), chlorophyll a (a), chlorophyll b (b), and superoxide dismutase (SOD), drought stress (DS), normal watering (CK), Coefficient of Variation (CV), different letters between control and treatment significant differences at *P* < 0.001 levels through paired sample t-test.*

### Drought Tolerance Is Explained by the Membership Function Value of Drought Resistance

**FIGURE 1:**
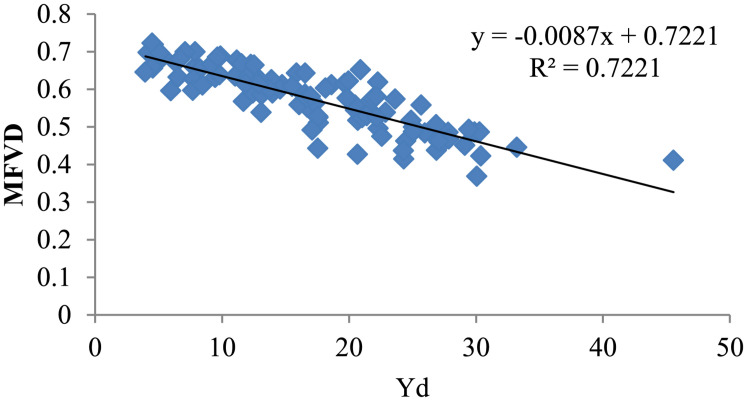
Linear regression analysis of the average value of MFVD and Yd in 3 years (MFVD, membership function value of drought resistance; Yd, yield reduction value).

Previous studies have shown that after water-limited treatment, the appropriate index is significantly correlated with yield. To discover a suitable water resistance index of varieties under drought conditions, the yield of 104 cotton varieties was measured under well-watered and water-limited conditions. MFVD is the average membership function value of drought resistance of all the target traits. The Yd value reflects the change in yield of cotton material under water stress. A lower Yd value corresponds to less yield reduction caused by drought stress and thus corresponds to stronger drought tolerance, while a higher Yd value indicates more yield reduction and weaker drought tolerance. Our statistics showed that after water stress in 2017, the yield reduction values of Xin lu zao 45, Xin lu zao 19, and Zhong R 2016 were the lowest (0.13 g/plant, 0.86 g/plant, and 0.06 g/plant; Appendix Table 4), respectively, while the MFVD values of Xin lu zao 45, Xin lu zao 19, and Zhong R 2016 were relatively large (0.73, 0.70, and 0.64, respectively; Table 3). Additionally in 2017, after cotton material resources were subjected to water stress, the yields of Xin lu zao 26, Xin hai 20 and Xin nong mian 3 decreased by 29.99 g/plant, 35.10 g/plant and 43.55 g/plant (Appendix Table 4), respectively, while the MFVD values were relatively lower (0.41, 0.39, and 0.46, respectively; Table 3). The same results were shown in 2016 and 2018 (a large Yd value means a lower MFVD value). The results showed that Yd has a linear relationship with MFVD and is significantly correlated in 3 years. The results showed that the MFVD can indicate the strength of drought tolerance (Figure 1).

**TABLE 3:** MFVD values, MFVD_1_ value and classification of some cotton varieties during 2016∼ 2018.

Varieties	2016		2016		2017		2017		2018		2018	
						
	MFVD	Group	MFVD_1_	Group	MFVD	Group	MFVD_1_	Group	MFVD	Group	MFVD_1_	Group
10599	0.43	III	0.52	II	0.53	III	0.52	III	0.35	III	0.42	III
108 Fu	0.70	II	0.70	II	0.57	III	0.66	II	0.51	II	0.60	II
2 Hao	0.38	IV	0.36	III	0.49	III	0.53	III	0.41	III	0.41	III
5917-N10-1	0.64	II	0.61	II	0.63	II	0.73	II	0.58	I	0.64	II
Xin lu Zao45	0.73	I	0.75	I	0.73	I	0.70	II	0.53	II	0.59	II
CQJ-5	0.64	II	0.54	II	0.66	II	0.68	II	0.63	I	0.66	II
KK1543	0.81	I	0.86	I	0.68	II	0.67	II	0.51	II	0.51	III
MSCO-12	0.76	I	0.84	I	0.71	I	0.88	I	0.51	II	0.61	II
ND359-5	0.77	I	0.78	I	0.68	II	0.89	I	0.58	I	0.56	II
TM-1	0.77	I	0.71	II	0.65	II	0.77	I	0.51	II	0.62	II
Bellsno	0.63	II	0.58	II	0.82	I	0.89	I	0.55	II	0.59	II
Xin hai 20	0.46	III	0.42	III	0.39	IV	0.32	IV	0.42	III	0.47	III
Shi yuan 321	0.57	III	0.53	II	0.61	II	0.68	II	0.50	II	0.63	II
Tai yuan 112	0.74	I	0.71	II	0.65	II	0.73	II	0.51	II	0.49	III
Tiao he 2013	0.72	II	0.64	II	0.76	I	0.85	I	0.67	I	0.75	I
Tian yun 10	0.64	II	0.56	II	0.61	II	0.66	II	0.56	II	0.53	III
Xi bu 50	0.56	III	0.48	II	0.44	IV	0.39	IV	0.46	III	0.48	III
Kui 85-174	0.49	III	0.34	III	0.46	IV	0.52	III	0.39	III	0.42	III
Xin lu zao 26	0.46	III	0.41	III	0.41	IV	0.40	IV	0.47	III	0.50	III
Xin lu zao 38	0.70	II	0.68	II	0.67	II	0.73	II	0.52	II	0.58	II
Xin lu zao 3	0.45	III	0.42	III	0.61	II	0.56	III	0.39	III	0.37	III
Xin pao 1 hao	0.46	III	0.42	III	0.66	II	0.68	II	0.44	III	0.47	III
Xin shi K7	0.48	III	0.19	IV	0.50	III	0.53	III	0.35	III	0.39	III
Xin lu Zao 13	0.73	I	0.82	I	0.79	I	0.87	I	0.65	I	0.64	II
Xin lu Zao 19	0.61	II	0.50	II	0.70	I	0.78	I	0.48	II	0.58	II
Xin lu Zao 32	0.44	III	0.35	III	0.43	IV	0.53	III	0.44	III	0.53	III
Xin lu Zao 7	0.59	II	0.56	II	0.73	I	0.75	II	0.49	II	0.62	II
Zhong R 2067	0.47	III	0.36	III	0.55	III	0.54	III	0.46	III	0.55	II
Zhong R 2016	0.75	I	0.59	II	0.64	II	0.68	II	0.44	III	0.46	III
Zhong R 773	0.60	II	0.58	II	0.68	II	0.69	II	0.57	II	0.63	II

### Identification of Key Drought Tolerance Indicators Through PCA and Stepwise Regression Analysis

PCA avoids repeated information interference without losing the original information by converting multiple indicators into new comprehensive and independent indicators. It can clearly display the changes in these indicators after stress. In our study, five top principal components were extracted, marked as PC1 to PC5, which together explained 65.52, 63.59, and 65.90% of the total variation after drought stress in 2016, 2017, and 2018, respectively (Table 4).

**TABLE 4:** Eigenvalues and contribution rate of principal components in 2016∼2018.

Principal components	2016		2017		2018	
			
	Eigenvalues	Cumulative contribution rate (%)	Eigenvalues	Cumulative contribution rate (%)	Eigenvalues	Cumulative contribution rate (%)
PC1	5.148	27.094	3.994	21.020	3.738	19.676
PC2	2.353	39.476	2.807	35.794	3.127	36.132
PC3	2.137	50.722	2.297	47.884	2.699	50.336
PC4	1.631	59.307	1.793	57.322	1.593	58.721
PC5	1.181	65.524	1.191	63.593	1.362	65.889

As shown in Table 5, the first principal component is mainly related to EFNB, BN and EBN, and these are morphological characteristics that can be defined as a comprehensive morphological index. The second principal component is related to photosynthetic traits such as Tr and Pn, and it can be defined as a comprehensive evaluation index of photosynthesis. The third main component is related to yield traits such as SBW, which can be defined as a comprehensive evaluation index of yield. The fourth main component is related to physiological traits such as Chl, and it can be defined as a comprehensive evaluation index of physiological traits. Additionally, PC5 is mainly Tr, indicating that it has a greater response to drought in photosynthetic indicators. In contrast to the results of 2016 and 2018, they are basically consistent with 2017 (Appendix Tables 2, 3). This finding indicates that we can use these five principal components to comprehensively evaluate cotton drought tolerance.

**TABLE 5:** Eigenvector matrix of principal component analysis.

	Principal component				
	
	PC1	PC2	PC3	PC4	PC5
PH	0.559	0.051	–0.329	–0.065	–0.040
FBN	0.493	0.190	–0.329	0.099	0.250
EFBN	0.814	–0.014	–0.403	0.158	0.115
BN	0.809	–0.099	–0.393	0.187	–0.128
EBN	0.804	–0.126	–0.387	0.176	–0.124
CSY	0.625	–0.320	0.534	–0.373	0.012
CLY	0.566	–0.266	0.421	–0.360	–0.052
SWB	0.625	–0.320	0.534	–0.373	0.012
CI	0.332	0.515	0.252	0.111	–0.182
gs	0.334	0.667	0.129	0.104	–0.119
VPD	0.141	0.671	0.063	0.059	–0.318
Pn	0.197	0.502	0.366	0.186	0.229
Tr	0.223	0.404	0.231	–0.071	0.591
WUE	0.079	0.693	0.391	0.004	–0.009
MDA	–0.064	0.064	–0.136	–0.221	–0.250
a	0.122	–0.498	0.317	0.439	0.230
b	0.026	–0.081	0.365	0.578	–0.387
Chl	0.095	–0.375	0.417	0.764	–0.017
SOD	–0.080	0.104	–0.132	0.237	0.514

At present, relevant literature has reported a large number of drought tolerance indicators in crops. Thus, it is necessary and urgent to screen out important indicators to accurately and rapidly select drought-tolerance varieties. In this study, stepwise regression analysis was performed to select appropriate indicators to comprehensively assess the drought tolerance of the cotton varieties based on the MFVD and the 19 trait indices. The MFVD value and 19 trait indicators were set as the dependent and independent variables, respectively. In 2017, the best regression equation *y* = (−0.307+0.142X_1_+0.419X_2_+0.355X_3_+0.091X_4_+0.120X_5_) was obtained through stepwise regression analysis. In the equation, X_1_, X_2_, X_3_, X_4_, and X_5_ represent five traits, namely, PH, EFBN, SBW, Tr, and Chl, whose coefficients were 0.142, 0.419, 0.355, 0.091, and 0.120, respectively, and the determination coefficient R^2^ of the equation was 0.73. The results suggested that those 5 independent variables can determine 73% of the total variation, and the equation is significant. In the regression equations established in 2016 and 2018, *R*^2^ is 0.82 and 0.71, respectively (Table 6). It can be seen from the regression equation that these 5 trait indices have significant effects on the drought tolerance of cotton materials and can be used as key indicators for comprehensive evaluation.

**TABLE 6:** Regression equation during 2016∼2018.

Year	Regression equation	*R*^2^	Sig.
2016	*y* = −0.294+0.229X_1_+0.368X_2_+0.308X_3_+0.184X_4_+0.133X_5_	0.82	0.000**
2017	*y* = −0.307+0.142X_1_+0.419X_2_+0.355X_3_+0.091X_4_+0.120X_5_	0.73	0.000**
2018	*y* = −0.166+0.135X_1_+0.197X_2_+0.151X_3_+0.211X_4_+0.229X_5_	0.71	0.000**

**Indicate significance at *P* < 0.01 level.

*X_1_:PH,X_2_:EFBN,X_3_:SBW,X_4_:Tr,X_5_:Chl.*

### Evaluation of Drought Tolerance in Cotton by MFVD

Cluster analysis results of 104 cotton varieties based on 19 indices show that the highest MFVD was observed in Zhong R 2016 (0.64) and Xin lu zao 45 (0.73) in 2017, which confirmed that the yields of Zhong R 2016 and Xin lu zao 45 decreased the least and were highly tolerant to drought stress. Furthermore, Xin lu zao 26 (0.41) and Xin hai 20 (0.39) had the lowest MFVD, indicating that the yield of these two cotton varieties exhibited the greatest decrease and highest sensitivity to drought stress. The same situation was also observed in 2016 and 2018 (Table 3).

Interestingly, in 2017 calculating the MFVD_1_ value based on these 5 indicators suggested that among 104 cotton varieties, Xin lu zao 45 and Zhong R 2016 still had high MFVD_1_ values of 0.70 and 0.68, respectively. Xin lu zao 26 and Xin hai 20 also have very low MFVD_1_ values of 0.40 and 0.32, respectively (Table 3). The analysis results from 2016 and 2018 are the same. High MFVD_1_ values have characteristics of strong drought tolerance, and low MFVD1 values are sensitive to drought tolerance. These results signify that these 5 traits are key drought tolerance indices in cotton.

## Discussion

Drought stress has a significant effect on morphology, and water stress reduces plant performance in all aspects, such as morphology, physiological characteristics, and yield (Claeys and Inze’D, 2013). Many scholars have conducted drought resistance identification from one or two aspects of morphology, photosynthesis and physiology because yield is affected by growth and development processes, and plant growth is a measure of drought adaptability; thus, the measured indicators must include yield and morphological indicators (Blum, 1979; Dolferus, 2014). Most studies have evaluated the drought resistance of cotton materials through morphological indicators and yield indicators (Liu et al., 2016; Li et al., 2017). Moreover, these indicators are only partial indicators and ignore the effects of photosynthesis, physiology and biochemistry on crop drought resistance. Osmotic adjustment is considered an important part of drought resistance (such as MDA and proline content; Wei et al., 2009; Fang and Xiong, 2015; Chen et al., 2016), and there is a positive correlation with the indicators of photosynthesis (Hura et al., 2007). At the same time, drought stress will cause the decomposition of Chl, which in turn affects crop photosynthesis (Efeoglu et al., 2009; Ying et al., 2015). Therefore, it is necessary to combine multiple traits such as morphology, physiology, biochemical and yield traits when conducting drought resistance evaluation and analysis. In our experiment, a total of 104 materials and 19 indices closely related to drought tolerance, including PH, FBN, EFBN, BN, EBN, CSY, CLY, SBW, Pn, gs, Tr, WUE, Ci, VPD, MDA, Chl, a, b, and SOD, and these data were used to screen the key indicators for evaluating drought tolerance in cotton. We found that their drought tolerance was different and distributed in different drought tolerance categories. The results of the 3-year analysis of variance showed that all traits had extremely significant differences after water treatment. Simultaneously, most of the CV values under drought stress were higher than that of the control, indicating that the cotton varieties types selected in this study are abundant, the treatment effect is obvious, and the results are representative.

The drought tolerance mechanism of plants is very complicated, and a single indicator cannot fully and accurately evaluate drought tolerance. Therefore, it is necessary to evaluate the drought tolerance of plants’ comprehensive character index by using a multivariate analysis method. PCA can simplify multiple variables by transforming the number of associated traits into a smaller number of representative variables as principal components (PCs). In rice, corn and wheat, PCA is used for drought resistance analysis and evaluation, and relevant drought resistance indicators have been determined. PCA can explain and describe the important indicators of drought resistance and salt tolerance in germplasm (Bo et al., 2017; Negrão et al., 2017; Kakar et al., 2019). Ayalew et al. (2011) identified three principal components through PCA, which accounted for 70% of the total variation in 14 agronomic traits. The main component PC1 shows that grain yield, biological yield and harvest indicators are closely related. This shows that the yield traits of crops are sensitive to drought stress, and there are large differences among varieties. Bedane et al. (2015) found that 73% of the 11 traits can be explained by two dimensions (PC1 and PC2). He found that PC1 is mainly the three indicators of plant height, ear length and biomass, and PC2 is mainly the number of tillers per plant and grain yield. In this study, similar results were obtained for cotton boll weight and yield traits per plant (with extremely significant differences among varieties after water treatment). In the abovementioned studies, the indicators for PCA analysis are all morphological indicators, which pay too much attention to morphological aspects while ignoring other aspects. PCA screened out important relevant indicators unilaterally in physiological indicators. Two important indicators of PF parameters have been determined through 18 indicators of chlorophyll fluorescence, which can screen many samples for large-scale surveys in a short time (Filippo et al., 2020). However, these indicators are based only on physiological indicators. In research on the drought resistance of irises, PCA is combined with regression analysis to screen out the water loss rate, and the activity of MDA and peroxidase can be used as important indicators for drought resistance evaluation (Bo et al., 2017). Although regression analysis is combined in the process of iris drought resistance research, only physiological indicators are analyzed, and other indicators are not analyzed. There are also a large number of drought tolerance evaluation indicators in cotton (including some of the indicators employed in the above research), which can also be effectively selected by these two methods. During the 3 years of this study, combined with PCA (five principal components were identified), through stepwise regression analysis, it was determined that the five traits (PH, EFBN, SBW, Tr, and Chl) had significant effects on the drought tolerance of cotton materials and could be used as the main indicators for screening drought-tolerance materials. With the development of remote sensing technology, new research methods have been provided for field research on large groups of crops. In agriculture, remote sensing technology is currently mainly used in research on crop diseases and insect pests (Calderon et al., 2013; Jan et al., 2015; Ballester et al., 2017), vegetation coverage (Li et al., 2012; Liu F. et al., 2014), and crop yield estimation (Tamouridou et al., 2017; Zhou et al., 2017). Combining remote sensing technology has been used in the detection and evaluation of potato late blight (Rodríguez et al., 2021), the monitoring of wheat yellow rust (Guo et al., 2021), and the estimation of tomato yield (Chang et al., 2021). But the application of remote sensing in cotton is less (but it has begun to develop in recent years). When remote sensing is used for large-scale and rapid measurements, some clear indicators are needed for analysis, such as the yield indicators and disease resistance indicators mentioned above. In this study, through the screening of cotton drought tolerance indicators, five key indicators reflecting cotton drought tolerance were initially determined, which provided preliminary target traits for the application of remote sensing in cotton, and these target traits can be used as reference parameters. This will provide an effective method and index for the large-scale assessment of drought tolerance in cotton varieties.

A single index can only reflect the sensitivity of a certain trait to drought during stress but cannot effectively reflect the comprehensive performance of crops under drought stress. MFVD is a multivariate index in which multiple traits are used to calculate its value. The MFVD value integrates drought resistance coefficients of different traits (Nouri-Ganbalani et al., 2009; Liu et al., 2015). It can effectively reflect the comprehensive performance of crops under drought stress. In wheat, the membership function is used for the identification and evaluation of drought resistance. Wheat materials with strong drought resistance, which are useful for drought resistance breeding, have been screened using MFVD (Chen et al., 2012; Liu et al., 2015; Song et al., 2017). However, none of the tested varieties exceeded 90, and the measured indicators were all morphological indicators such as PH, FLA (area of flag leaf), etc., and no comprehensive consideration was given to the selection of relevant indicators. In this study, the drought tolerance of cotton materials was evaluated by membership function. The MFVD values of the nineteen indicators classify the tested cotton materials into four types, and the MFVD_1_ of the five indicators screened by PCA are also divided into four categories. In 3 years, MFVD and MFVD_1_ showed a very significant positive correlation, with correlation coefficients of 0.889, 0.829, and 0.841, respectively (*p* < 0.01). Three-year analysis results show that this method can increase the accuracy of drought tolerance evaluation in cotton fields. The five indicators screened by PCA can be used for the identification of cotton drought tolerance and the screening of drought-resistant materials.

## Conclusion

In this experiment, 19 drought-related indicators such as morphology, photosynthesis, physiology and yield were measured after water stress, and five main components were identified through PCA to effectively explain the drought tolerance of cotton. These five indices, including PH, EFBN, SBW, Tr and Chl, were selected in combination with stepwise regression analysis. The MFVD values of 19 indicators are basically consistent with the evaluation results of the MFVD_1_ values obtained from the five indicators. Eventually, these five indicators were selected as the key indicators to evaluate the drought tolerance of cotton. These findings will help us evaluate drought tolerance rapidly and subsequently and then screen drought-tolerance materials.

## Data Availability Statement

The original contributions presented in the study are included in the article/Supplementary Material, further inquiries can be directed to the corresponding author/s.

## Author Contributions

YQ designed and supervised the experiment. FS conducted experiment, analyzed the data, and drafted the manuscript. QC revised the manuscript. QJC and WG provided ideas. MJ helped with measurement and statistics.

## Conflict of Interest

The authors declare that the research was conducted in the absence of any commercial or financial relationships that could be construed as a potential conflict of interest.

## References

[B1] Ali M. N. Yeasmin L. Gantait S. Goswami R. Chakraborty S. (2014). Screening of rice landraces for salinity tolerance at seedling stage through morphological and molecular markers. *Physiol. Mol. Biol. Plants* 20 411–423. 10.1007/s12298-014-0250-6 25320465 PMC4185050

[B2] Ayalew H. Genet T. Dessalegn T. Wondale L. (2011). Multivariate diversity, heritability and genetic advance in tef landraces in Ethiopia. *Afr. Crop Sci. J*. 19 201–212.

[B3] Ballester C. Hornbuckle J. Brinkhoff J. Smith J. Quayle W. (2017). Assessment of in-season cotton nitrogen status and lint yield prediction from unmanned aerial system imagery. *Remote Sens.* 9 1149–1166. 10.3390/rs9111149

[B4] Bedane G. M. Saukuru A. M. George D. L. Gupta M. L. (2015). Evaluation of tef (Eragrostis tef [Zucc.] Trotter) lines for agronomic traits in Australia. *Aust. J. Crop Sci.* 9 242–247.

[B5] Blum A. (1979). “Genetic improvement of drought resistance in crop plants: a case for sorghum,” in *Stress Physiology in Crop Plants*, eds Mussell H. Stables R. C. (New York, NY: John Wiley and Sons Inc), 429–445.

[B6] Blum A. Jordan W. R. (1985). Breeding crop varieties for stress environments. *Crit. Rev. Plant Sci.* 2 199–238. 10.1080/07352688509382196

[B7] Bo W. Fu B. C. Qin G. J. Xing G. M. Wang Y. G. (2017). Evaluation of drought resistance in Iris germanica L. based on subordination function and principal component analysis. *EJFA* 29 770–778. 10.9755/ejfa.2017.v29.i10.1260

[B8] Calderon R. Navas-Cortes J. A. Lucena C. Zarco-Tejada P. J. (2013). High-resolution airborne hyperspectral and thermal imagery for early detection of verticilliumwilt of olive using fluorescence, temperature and narrow-band spectral indices. *Remote Sens. Environ*. 139 231–245. 10.1016/j.rse.2013.07.031

[B9] Cattivelli L. Rizza F. Badeck F. W. Mazzucotelli E. Mastrangelo A. M. Francia E. (2008). Drought tolerance improvement in crop plants: an integrated view from breeding to genomics. *Field Crops Res.* 105 1–14. 10.1016/j.fcr.2007.07.004

[B10] Chang A. J. Jung J. H. Yeom J. Maeda M. M. Landivar G. A. Enciso J. M. (2021). Unmanned aircraft system- (UAS-) based high-throughput phenotyping (HTP) for tomato yield estimation. *J. Sens*. 2021:8875606. 10.1155/2021/8875606

[B11] Chen D. Neumann K. Friedel S. Kilian B. Chen M. Altmann T. (2014). Dissecting the phenotypic components of crop plant growth and drought responses based on high-throughput image analysis. *Plant Cell* 26 4636–4655. 10.1105/tpc.114.129601 25501589 PMC4311194

[B12] Chen D. Q. Wang S. W. Li H. B. Yin L. A. Cao B. B. Shan L. (2016). Genotypic variation in growth and physiological response to drought stress and re-watering reveals the critical role of recovery in drought adaptation in maize seedlings. *Front. Plant Sci.* 6:1241. 10.3389/fpls.2015.01241 26793218 PMC4709455

[B13] Chen X. J. Min D. H. Yasir T. A. Hu Y. G. (2012). Evaluation of 14 morphological, yield-related and physiological traits as indicators of drought tolerance in Chinese winter bread wheat revealed by analysis of the membership function value of drought tolerance (MFVD). *Field Crop Res*. 137 195–201. 10.1016/j.fcr.2012.09.008

[B14] Claeys H. Inze’D D. (2013). The agony of choice: how plants balance growth and survival under water-limiting conditions. *Plant Physiol.* 162 1768–1779. 10.1104/pp.113.220921 23766368 PMC3729759

[B15] Dolferus R. (2014). To grow or not to grow: a stressful decision for plants. *Plant Sci*. 229 247–261. 10.1016/j.plantsci.2014.10.002 25443851

[B16] Du X. M. Zhou Z. L. (2005). *Description Specifications and Data Standards for Cotton Germplasm Resources.* Beijing: China Agriculture Press.

[B17] Efeoglu B. Ekmekci Y. Cicek N. (2009). Physiological responses of three maize cultivars to drought stress and recovery. *South Afr. J. Bot*. 75 34–42. 10.1016/j.sajb.2008.06.005

[B18] Fang Y. Xiong L. (2015). General mechanisms of drought response and their application in drought resistance improvement in plants. *Cell. Mol. Life Sci*. 72 673–689. 10.1007/s00018-014-1767-0 25336153 PMC11113132

[B19] Farshadfar E. Elyasi P. Aghaee M. (2011). In Vitro selection for drought tolerance in common wheat (*Triticum aestivum L.*) genotypes by mature embryo culture. *Am. J. Sci. Res*. 48 102–115.

[B20] Filippo B. Giacomo G. Anthony D. Martina P. (2020). Selection of chlorophyll fluorescence parameters as indicators of photosynthetic efficiency in large scale plant ecological studies. *Ecol. Indic*. 108 1–10.

[B21] Flexas J. Bota J. Galmés J. Medrano H. Ribas-Carbó M. (2006). Keeping a positive carbon balance under adverse conditions: responses of photosynthesis and respiration to water stress. *Physiol. Plant* 127 343–352. 10.1111/j.1399-3054.2006.00621.x

[B22] Füzy A. Kovács R. Cseresnyés I. Parádi I. Szili-Kovács T. Kelemen B. (2019). Selection of plant physiological parameters to detect stress effects in pot experiments using principal component analysis. *Acta Physiol. Plant* 41 1–10.

[B23] Golestani-Araghi S. Assad M. T. (1998). Evaluation of four screening techniques for drought resistance and their relationship to yield reduction ratio in wheat. *Euphytica* 103 293–299.

[B24] Guo A. Huang W. Dong Y. Ye H. C. Ma H. Q. Liu B. (2021). Wheat yellow rust detection using UAV-based hyperspectral Technology. *Remote Sens*. 13:123. 10.3390/rs13010123

[B25] Hura T. Grzesiak S. Hura K. Thiemt E. Tokarz K. Wedzony M. (2007). Physiological and biochemical tools useful in drought-tolerance detection in genotypes of winter triticale: accumulation of ferulic acid correlates with drought tolerance. *Ann. Bot*. 100 767–775. 10.1093/aob/mcm162 17684022 PMC2749628

[B26] Huseynova I. M. Rustamova S. M. Suleymanov S. Y. Aliyeva D. R. Mammadov A. C. Aliyev J. A. (2016). Drought-induced changes in photosynthetic apparatus and antioxidant components of wheat (*Triticum durum* Desf.) varieties. *Photosynth. Res*. 130 215–223. 10.1007/s11120-016-0244-z 26988099

[B27] Hussein F. Janat M. Yakob A. (2011). Assessment of yield and water use efficiency of drip-irrigated cotton (*Gossypium hirsutum L.*) as affected by deficit irrigation. *Turkish J. Agric. For.* 35 611–621.

[B28] Jan L. Felix N. Torsten P. Christian K. (2015). Analysis of unmanned aerial system-based CIR images in forestry – a new perspective to monitor pest in – festation levels. *Forests* 6 594–612. 10.3390/f6030594

[B29] Kakar N. Jumaa S. H. Redoña E. D. Warburton M. L. Reddy K. R. (2019). Evaluating rice for salinity using pot-culture provides a systematic tolerance assessment at the seedling stage. *Rice* 57 1–14. 10.4038/tar.v31i2.8362 PMC6667605 31363935

[B30] Kang Y. H. Wang R. S. Wan S. Q. Hu W. Jiang S. F. Liu S. P. (2012). Effects of different water levels on cotton growth and water use through drip irrigation in an arid region with saline ground water of Northwest China. *Agr. Water Manage*. 109 117–126. 10.1016/j.agwat.2012.02.013

[B31] Khalili M. Naghavi M. R. Aboughadareh A. P. Talebzadeh S. J. (2012). Evaluating of drought stress tolerance based on selection indices in spring canola cultivars (*Brassica napus L.*). *JAS* 4 78–85.

[B32] Kramer D. M. Evans J. R. (2011). The importance of energy balance in improving photosynthetic productivity. *Plant Physiol*. 155 70–78. 10.1104/pp.110.166652 21078862 PMC3075755

[B33] Lawlor D. W. Cornic G. (2002). Photosynthetic carbon assimilation and associated metabolism in relation to water deficit in higher plants. *Plant Cell Environ*. 25 272–294. 10.1046/j.0016-8025.2001.00814.x 11841670

[B34] Li B. Liu R. Y. Liu S. H. Liu Q. Liu F. Zhou G. Q. (2012). Monitoring vegetation coverage variation of winter wheat by low-altitude UAV remote sensing system. *Trans. Chin. Soc. Agric. Eng*. 28 160–165.

[B35] Li Z. B. Zhang J. Wei Y. N. Yu J. Xi Z. L. Zhang X. J. (2011). Characteristics analysis of yield traits and fiber quality with cotton drought resistance under plastic film mulching and high-density condition. *J. Nuclear Agric. Sci*. 25 0576–0581.

[B36] Li Z. W. Chen Y. L. Luo J. J. Shi Y. T. Feng K. Y. Chen Z. X. (2017). Screening and evaluation for drought resistance of cotton varieties. *Agric. Res. Arid Areas* 35 240–247.

[B37] Lichtenthaler H. K. (1987). Chlorophylls and carotenoids: pigments of photosynthetic biomembranes. *Method Enzymol*. 148 350–382. 10.1016/0076-6879(87)48036-1

[B38] Liu C. Y. Yang Z. Y. Hu Y. G. (2015). Drought resistance of wheat alien chromosome addition lines evaluated by membership function value based on multiple traits and drought resistance index of grain yield. *Field Crops Res*. 179 103–112. 10.1016/j.fcr.2015.04.016

[B39] Liu F. Liu S. H. Xiang Y. (2014). Study on monitoring fractional vegetation cover of garden plots by unmanned aerial vehicles. *Trans. Chin. Soc. Agric. Machinery* 45 250–257.

[B40] Liu G. H. Chen Q. J. Wu P. H. Qu Y. Y. Gao W. W. Yang J. S. (2016). Screening and comprehensive evaluation of drought resistance indices of cotton at blossing and boll-forming stages. *J. Plant Genet. Resour*. 17 53–62.

[B41] Liu T. P. Dong K. J. He J. H. Ren R. Y. Zhang L. Yang T. Y. (2014). Identification and evaluation on the drought resistance of broomcorn millet bred cultivars at germinating stage. *J. Plant Genet. Resour*. 15 746–752.

[B42] Loka D. A. Oosterhuis D. M. Ritchie G. L. (2011). “Water-deficit stress in cotton [M],” in *Stress Physiology in Cotton, Number Seven The Cotton Foundation Book Series*, Vol. 2011 ed. Oosterhuis D. M. (Cordova, TN: National Cotton Council of America), 37–72.

[B43] Lv X. L. Bai H. B. Hui J. Tian X. Y. Yang C. G. Ma S. S. (2019). Evaluation of seedling drought resistance of RIL derived from indica rice and japonica rice. *J. Plant Genet. Resour*. 20 556–563.

[B44] Munir S. Qureshi M. K. Shahzad A. N. Nawaz I. Anjam S. Rasul S. (2020). Genetic dissection of interspecific and intraspecific hybrids of cotton for morpho-yield and fiber traits using multivariate analysis. *Pak. J. Agric. Res*. 33 9–16.

[B45] Nagy Z. Németh E. Guóth A. Bona L. Wodala B. Pécsváradi A. (2013). Metabolic indicators of drought stress tolerance in wheat: Glutamine synthetase isoenzymes and Rubisco. *Plant Physiol. Bioch*. 67 48–54. 10.1016/j.plaphy.2013.03.001 23542183

[B46] Negrão S. Schmöckel S. M. Tester M. (2017). Evaluating physiological responses of plants to salinity stress. *Ann. Bot*. 119 1–11. 10.1093/aob/mcw191 27707746 PMC5218372

[B47] Nouri-Ganbalani A. Nouri-Ganbalani G. Hassanpanah D. (2009). Effects of drought stress condition on the yield and yield components of advanced wheat genotypes in Ardabil, Iran. *J. Food Agric. Environ*. 7 228–234.

[B48] Ramirez P. Kelly J. (1998). Traits related to drought resistance in common bean. *Euphytica* 99 127–136.

[B49] Rodríguez J. Lizarazo I. Prieto F. Angulo-Morales V. (2021). Assessment of potato late blight from UAV-based multispectral imagery. *Comput. Electron. Agric*. 184:106061. 10.1016/j.compag.2021.106061

[B50] Sarwar M. K. S. Ashraf M. Y. Rehman M. Zafar Y. (2012). Genetic variability in different biochemical traits and their relationship with yield and parameters of cotton cultivar grown under water stress conditions. *Pak. J. Bot*. 44 515–520.

[B51] Song Q. H. Liu C. Y. Bachir D. G. Chen L. Hu G. Y. (2017). Drought resistance of new synthetic hexaploid wheat accessions evaluated by multiple traits and antioxidant enzyme activity. *Field Crops Res*. 210 91–103. 10.1016/j.fcr.2017.05.028

[B52] Soomroo M. H. Markhand G. S. Soomro B. A. (2011). Screening Pakistani cotton for drought tolerance. *Pak. J. Bot*. 44 383–388.

[B53] Stackpole D. J. Vaillancourt R. E. Alves A. Rodrigues J. Potts B. M. (2011). Genetic variation in the chemical components of Eucalyptus globulus wood. *G3 (Bethesda)* 1 151–159. 10.1534/g3.111.000372 22384327 PMC3276126

[B54] Szira F. Balint A. Börner A. Galiba G. (2008). Evaluation of Drought-Related traits and screening methods at different developmental stages in spring barley. *J. Agron. Crop Sci*. 194 334–342. 10.1111/j.1439-037x.2008.00330.x

[B55] Tamouridou A. A. Alexandridis T. Pantazi X. E. Lagopodi A. L. Kashefi J. Moshou D. (2017). Evaluation of UAV imagery for mapping *Silybummarianum* weed patches. *Int. J. Remote Sens*. 38 2246–2259. 10.1080/01431161.2016.1252475

[B56] Ullah A. Sun H. Yang X. Zhang X. (2017). Drought coping strategies in cotton: in-creased crop per drop. *Plant Biotech. J*. 15 271–284. 10.1111/pbi.12688 28055133 PMC5316925

[B57] Wang C. Isoda A. Wang P. (2004). Growth and yield performance of some cotton cultivars in Xinjiang, China, an arid area with short growing period. *J. Agron. Crop Sci*. 190 177–183. 10.1111/j.1439-037x.2004.00090.x

[B58] Wang Z. M. He Y. J. Jin M. G. Wang B. G. (2010). Optimization of mulched drip-irrigation with brackish water for cotton using soil-water-salt numerical simulation. *Trans. Chin. Soc. Agric. Eng*. 28 63–70.

[B59] Wei L. Zhang D. Xiang F. Zhang Z. (2009). Differentially expressed miRNAs potentially involved in the regulation of defense mechanism to drought stress in maize seedlings. *Int. J. Plant Sci*. 170 979–989. 10.1086/605122

[B60] Wiggins M. S. Leib B. G. Mueller T. C. Main C. L. (2013). Investigation of physiological growth, fiber quality, yield, and yield stability of upland cotton varieties in differing environments. *J. Cotton Sci.* 17 140–148.

[B61] Wijewardana C. Henry W. B. Hock M. W. Reddy K. R. (2016). Growth and physiological trait variation among corn hybrids for cold tolerance. *Can. J. Plant Sci.* 96 639–656. 10.1139/cjps-2015-0286 33356898

[B62] Yang J. Zhang X. Peng Y. Huang L. Liang X. Wang K. (2014). Osmolyte accumulation, antioxidant enzyme activities and gene expression patterns in leaves of orchardgrass during drought stress and recovery. *Grassl. Sci*. 60 131–141.

[B63] Yin L. Wang S. Eltayeb A. E. Uddin M. I. Yamamoto Y. Tsuji W. (2010). Overexpression of dehydroascorbate reductase, but not monodehydroascorbate reductase, confers tolerance to aluminum stress in transgenic tobacco. *Planta* 231 609–621. 10.1007/s00425-009-1075-3 19960204

[B64] Ying Y. Q. Song L. L. Jacobs D. F. Mei L. Liu P. Jin S. H. (2015). Physiological response to drought stress in *Camptotheca acuminata* seedlingsfromtwo provenances. *Front. Plant Sci*. 6:361. 10.3389/fpls.2015.00361 26052334 PMC4440367

[B65] Zadeh L. A. (1965). Fuzzy sets. *Inf. Contr*. 8 338–353.

[B66] Zhang F. Wang Y. Lou Z. Dong J. (2007). Effect of heavy metal stress on antioxidative enzymes and lipid peroxidation in leaves and roots of two mangrove plant seedlings (*Kandelia candel* and *Bruguiera gymnorrhiza*). *Chemosphere* 67 44–50. 10.1016/j.chemosphere.2006.10.007 17123580

[B67] Zhang Y. L. Zhang H. Z. Du M. W. Li W. Luo H. H. Chow W. S. (2010). Leaf wilting movement can protect water-stressed cotton (*Gossypium hirsutum L.*) plants against photoinhibition of photosynthesis and maintain carbon assimilation in the field. *J. Plant Biol*. 53 52–60. 10.1007/s12374-009-9085-z

[B68] Zhou X. Zheng H. B. Xu X. Q. He J. Y. Ge X. K. Yao X. (2017). Predicting grain yield in rice using multi-temporal vegetation indices from UAV-based multispectral and digital imagery. *ISPRS J. Photogramm. Remote Sens*. 130 246–255. 10.1016/j.isprsjprs.2017.05.003

