# Supplementary material for: Screening of Key Drought Tolerance Indices for Cotton at the Flowering and Boll Setting Stage Using the Dimension Reduction Method
Source: Front Plant Sci. 2021 Jul 9;12:619926. doi: 10.3389/fpls.2021.619926 (PMC8299416; doi:10.3389/fpls.2021.619926)
Supplement: Supplementary file 2 [file Data_Sheet_2.docx]

APPENDIX 1

Determination of the Malondialdehyde (MDA) Content

The method for measuring the content of MDA was based on that reported by Yin et al. (2010). The leaf material (0.5 g) was homogenized in 5 ml of 0.1% TCA solution. The homogenate was centrifuged at 12,000 g for 20 min, and 0.5 ml of the supernatant was added to 1 ml 0.5% TBA in 20% TCA. The mixture was incubated in boiling water for 30 min; the reaction was terminated by placing the reaction tube in an ice bath. The sample was then centrifuged at 10,000 g for 5 min, and the supernatant was used forMDA analysis.

Determination of the Chlorophyll (Chl) Content

The Chl content was determined according to the methods described by Lichtenthaler (1987) and Yang et al. (2014). First 80% acetone was used to extract the pigment from the leaf sample (approximately 0.2 g) on a shaker until the tissue was completely bleached. The extract was centrifuged at 5000 g for 5 min, and the supernatant was collected for absorption. The measurement was performed at 646, 470, and 663 nm using a spectrophotometer (UV-2550, Shimadzu, Japan), and the Chl content was then calculated.

Determination of Superoxide Dismutase Enzyme Activity

The enzyme extract was mixed with the reaction solution (the mixture includes 50 mM phosphate buffer (pH 7.8), 0.1 mM EDTA, 13 mM methionine, 75 µM NBT, 2 µM riboflavin), and the reaction was started under two 15-W fluorescent lamps. The mixture solution was then placed without the supernatant at the same position. The same solution was stored as a blank solution in the dark. Lighting was stopped after 10 min (t). The absorbance of the blank at 560 nm was then measured, and the activity of SOD was calculated.
